# Consequences for Piglet Performance of Group Housing Lactating Sows at One, Two, or Three Weeks Post-Farrowing

**DOI:** 10.1371/journal.pone.0156581

**Published:** 2016-06-03

**Authors:** Ola Thomsson, Ylva Sjunnesson, Ulf Magnusson, Lena Eliasson-Selling, Anna Wallenbeck, Ann-Sofi Bergqvist

**Affiliations:** 1 Division of Reproduction, Department of Clinical Sciences, Swedish University of Agricultural Sciences, SLU, Uppsala, Sweden; 2 Farm & Animal Health, Uppsala, Sweden; 3 Department of Animal Breeding and Genetics, Swedish University of Agricultural Sciences, SLU, Uppsala, Sweden; University of British Columbia, CANADA

## Abstract

Housing lactating sows with piglets in a multi-suckling pen from around 14 days post-farrowing is common practice in Swedish organic piglet production. However, nursing-suckling interaction is less frequent in multi-suckling pens than in individual farrowing pens, thus affecting piglet performance, e.g., piglet growth. Moreover, piglet mortality is higher in systems using multi-suckling pens. Three management routines whereby lactating sows with piglets were moved from individual farrowing pens to multi-suckling pens at one, two, or three weeks post-farrowing were compared in terms of nursing-suckling interaction and piglet performance. Correlations between nursing-suckling interaction, piglet performance, and piglet mortality were also examined. In total, 43 Yorkshire sows with piglets were included in the study. Nursing-suckling interaction and all piglet performance parameters except piglet mortality did not differ between management routines. Piglet mortality in the individual farrowing pens did not differ between management routines, but piglet mortality in the multi-suckling pen was lower (*P*<0.05*)* when piglets were group housed at three weeks compared with one week post-farrowing. Overall piglet mortality was positively correlated with mortality in the multi-suckling pen for piglets group housed at one week (r = 0.61: *P*<0.05) and at two weeks post-farrowing (r = 0.62: *P*<0.05) but not for piglets group housed at three weeks post-farrowing. In conclusion, overall piglet mortality could be reduced if sows and piglets are group housed at three weeks post-farrowing and piglet survival the first week post-farrowing is improved.

## Introduction

Organic piglet production in Sweden is characterized by sows farrowing untethered in individual pens, followed by group housing of sows and piglets in a multi-suckling pen around 14 days post-farrowing and weaned at six weeks post-farrowing [1, 2]. However, sows housed in multi-suckling pens may avoid their piglets, leading to less frequent nursing-suckling interaction compared with in individual farrowing pens [3, 4]. Less frequent nursing-suckling interaction affects piglet performance, e.g., piglet growth [5]. Another factor related to nursing-suckling interaction and piglet performance is within-litter weight variation at weaning, as it is related to the sow’s ability to evenly nurse the piglets [6]. A large within-litter weight variation at weaning increases the labor requirement, e.g., sorting of piglets, and complicates management, e.g., piglets with different weights differ in feed requirements [7]. Most piglet mortality occurs within the first week post-farrowing [8]. However, overall piglet mortality is higher in production systems using multi-suckling pens than in systems with individual farrowing pens [9, 10]. The main cause of late death of live-born piglets is crushing by the sow, followed by starvation [11–13]. It has been suggested that the crushing could be an effect of the piglet being weakened because of missed nursing opportunities and lack of energy to escape crushing by the sow [14, 15].

The aims in the present study were thus to investigate: i) whether there were differences in nursing-suckling interaction between three management routines (grouping at one (W1), two (W2), or three weeks (W3) post-farrowing); and ii) whether possible differences in nursing-suckling interaction are associated with piglet performance (piglet weaning weight, within-litter weight variation, and piglet mortality).

## Materials and Methods

### Animals and experimental design

The study was conducted over the three-month period (September 7-December 14, 2011). Mean outdoor temperature decreased from 13°C in September to 2°C in December [16]. The study was performed at the Swedish Livestock Research Centre at Funbo-Lövsta, Uppsala, and was approved by the Uppsala Animal Ethics Committee (Approval C154/11).

The animals and the experimental design have been described in detail previously [17] and are briefly summarized here. Forty-three Yorkshire sows of parity one through nine were included in the experimental part of the study. One week before expected farrowing, sows and gilts were moved from a group gestation pen to straw bedded individual farrowing pens (7.6 m^2^) in which the sows were untethered. The individual farrowing pens were equipped with rails along the walls and a separate piglet creep area. The sows spent one (W1), two (W2), or three weeks (W3) in the individual farrowing pens post-farrowing, before being moved to a multi-suckling pen. Each management routine was repeated once (batch I and batch II). In batch I, 8 sows were included in management routine W1, 5 sows in W2, and 8 sows in W3. In batch II, 6 sows were included in management routine W1, 7 sows in W2, and 8 sows in W3. In total, 14 sows were included in W1; 13 sows in W2, and 16 sows in W3. Within each management routine and batch (1 and 2), sows were assigned to two subsets. The first subset comprised the first sows that farrowed within management routine and batch, and were the first to be moved to the multi-suckling pen (referred to as early, range 3–6 sows). The second subset of sows farrowed later, and these were moved one or three days later (referred to as late, range 2–5 sows). The farrowing interval ranged from 0–4 days for all management routines. Mean number of piglets per sow at the time of moving from the individual farrowing pen to the multi-suckling pen was 10.0 ± 2.1. All sows were weaned at six weeks (44 ± 1.6 days) post-farrowing. Thus, W1 sows spent five weeks in the multi-suckling pen, W2 sows spent four weeks, and W3 sows spent three weeks in the multi-suckling pen before weaning.

An additional nine lactating sows served as a reference group. This group was untethered in individual farrowing pens during the whole lactation and weaned at five weeks according to Swedish animal welfare law, with the exception that they were assigned the same feeding regime as the other sows. For the reference group, data regarding piglet mortality from birth to weaning, piglet weaning weight, and within-litter weight difference at weaning were recorded. The reference group data represented that of the Swedish Livestock Research Centre in general.

### Housing

Three multi-suckling pens were constructed in an uninsulated barn. The pigs had no outdoor access. The space allowance per sows was according to Swedish organic standards (7.5 m^2^) [2]. Each multi-suckling pen was divided by a wall into two larger areas, referred to as a lying area and a feeding area. An opening in the dividing wall allowed sows and piglets to move back and forth between the two areas at all times. A piglet creep area equipped with a roof and four heating lamps was situated in one corner of the feeding area. In each multi-suckling pen, two water nipples with bowls underneath provided the sows and piglets with water *ad libitum*. Throughout their lactation, sows were fed *ad libitum* in a feeding trough in the feeding area with a standard commercial lactation feed (DIA 120; 12.8 MJ/kg; 160 crude protein/kg, Lantmännen, Sweden). The piglets were able to eat sow feed from the trough at all times. Grass hay was provided *ad libitum*. Straw was used as bedding material in the lying area and peat in combination with straw was used in the feeding area.

### Assessing nursing-suckling interaction

The nursing-suckling interaction was assessed from video recordings, using a modification of a protocol described previously [5, 18] (Table 1). Two multi-suckling pens were equipped with three infra-red sensitive cameras each, while the third pen had an additional fourth camera. The cameras recorded the entire group housing period. The cameras were mounted so that the entire pen area was captured.

**Table 1:** Activities related to the nursing-suckling interaction studied in video recordings and their definition.

Action	Definition
Start of nursing/suckling	≥5 piglets active at the udder
End of nursing	<5 piglets active at the udder or the sow rolls over from the side to sternal recumbency.
Termination	Piglet: If <5 piglets active at the udder Sow: If the sow rolls over from the side to sternal recumbency or stands up and walks away.

Each sow was individually marked with standard color spray on the back for identification on the video. Nursing-suckling interactions were recorded in weeks 4 and 6 of lactation in all groups. Due to a power failure, no video recordings were available during week 5 of lactation. In each recording week, two separate days, with two days in between, were used. From each recording day 7.5 hours of recordings were used from video recordings. These hours were between 9:00 and 16:30, during which no other activity in the pen interfered. The nursing-suckling interaction was recorded as nursing duration, nursing frequency, and duration per nursing event [5, 19, 20]. Nursing duration was calculated as the total time spent nursing per sow and day (7.5 h). Nursing frequency was the average number of nursings per sow and day (7.5 h). Duration per nursing was average duration per nursing event, based on all nursing events per sow and day (7.5 h). The nursing-suckling interaction was analyzed in three different datasets: 1) all nursings, 2) nursings terminated by the sow, and 3) nursings terminated by the piglets. Because no sound was recorded, it was not possible to determine whether the sow or the piglets initiated the nursing.

### Assessing piglet performance

Piglet performance was assessed at weaning by mean piglet weight per litter, within-litter weight variation, and mean litter weight. Mean piglet weight gain and litter weight gain from birth to weaning and piglet mortality were also included. Each piglet had a number tattooed on the right ear for identification and each piglet was weighed once a week from one week of age until the day of weaning. If a piglet was found dead or was euthanized, the following data were recorded: date, ID number, body weight, and assumed cause of death or reason for euthanization. For all piglets that died or were euthanized in the multi-suckling pen, a simple post-mortem examination was conducted where body condition (normal/thin) was determined and the stomach was examined for feed content.

### Statistical analysis

Statistical analyses were performed using the SAS software ver. 9.3 (SAS Inst. Inc., Cary, NC, USA). Residuals of all dependent y-variables analyzed in the five different models below were examined for normal distribution using PROC UNIVARIATE, considering the Shapiro-Wilks test for normality and a normal probability plot. All residual variables were found to be normally or approximately normally distributed.

Differences between management treatments in terms of nursing duration, nursing frequency, and duration per nursing event were analyzed using a mixed model in PROC MIXED (MODEL 1). Analyses were performed on three separate datasets; one including all nursing information, one including only sow-terminated nursings, and one including only piglet-terminated nursings.

MODEL 1:

y = management routine + batch + subset + management routine*batch + week of lactation+  management treatment*week of lactation + day in lactation (week of lactation) + sow (management treatment*batch) + e

In Model 1, management routine (W1, W2, and W3), batch (I and II), subset (early or late move), the interaction between management routine*batch, week of lactation (week 4 or 6), and the interaction between management routine and week of lactation were included as fixed class effects. Day in lactation nested within week of lactation was included as a continuous covariate and sow nested within the interaction between management treatment and batch was included as a random effect.

Differences between management routines in piglet growth, piglet weaning weight, and within-litter weight variation were analyzed using general linear models in PROC GLM (MODEL 2).

MODEL 2:

y = management routine + batch + subset + parity number + management routine*batch + litter birth weight + litter size at birth + e

In Model 2, management routine (W1, W2, and W3), batch (I or II), subset (early or late), a parity number (1, 2, and >2), and the interaction between management routine and batch were included as fixed class effects. Litter birth weight and litter size at birth (number of live-born piglets) were included as continuous covariates. When litter weight at weaning, litter growth from birth until weaning, and within-litter weight variation were analyzed, total litter weight at birth was included as a covariate, while mean litter birth weight was included when mean piglet weight at weaning and litter mean piglet growth were analyzed.

Differences between management routines in piglet mortality (total, before grouping, and after grouping) and litter size at weaning were analyzed using general linear models in PROC GLM (MODEL 3).

MODEL 3:

y = management routine + batch + subset +management routine*batch + parity number + management routine*batch + litter size at birth + e

In Model 3, management treatment (W1, W2, and W3), batch (I or II), subset (early or late), the interaction between management routine*batch, and parity number (1, 2, and >2) and the interaction between management routine and batch were included as fixed class effects and litter size at birth (number of live born piglets) was included as a covariate. When piglet mortality after grouping was analyzed, litter size at group housing was added as a covariate.

To assess differences between management routines (until week 5 after farrowing) and the reference group in terms of piglet weight, piglet growth, and within-litter weight variation, general linear models were used in PROC GLM (MODEL 4).

MODEL 4:

y = management routine + parity number + management routine*batch + litter weight at birth + litter size at birth + e

In Model 4, management treatment (W1, W2, W3, and reference group), parity number (1, 2, and >2), and the interaction between management routine and batch were included as fixed class effects and litter weight and litter size at birth (number of live born piglets) were included as continuous covariates. When total litter weight, litter growth and within-litter weight variation were analyzed, total litter birth weight was included as a covariate, while mean litter birth weight was included when mean piglet weight and growth were analyzed.

To assess differences between management routines (until week 5 post-farrowing) and the reference group in piglet mortality, general linear models were analyzed using in PROC GLM (MODEL 5).

MODEL 5:

y = management routine + parity number + litter size at birth + e

In Model 5, management treatment (W1, W2, W3, and reference group) and parity number (1, 2, and >2) were included as fixed class effects and litter size at birth (number of live-born piglets) was included as a covariate.

Associations between nursing behavior and piglet performance (weight, growth, and mortality) were investigated through residual Pearson correlations using PROC CORR with residuals from Models 1, 2, and 3. Correlations were estimated separately for the whole dataset and for each management routine separately. When nursing variables were analyzed, they were considered for each observation day in weeks 4 and 6 separately (two observation days per week).

## Results

### Nursing-suckling interaction

There were no significant differences between the three management routines with respect to total nursing duration per day, nursing frequency per day, or duration per nursing event when datasets of all nursings, sow-terminated, or piglet-terminated nursings were analyzed (Table 2).

**Table 2:** Total nursing duration per sow and day (s), average nursing frequency per sow and day (s), and average duration per nursing event per sow and day (s) for the three management routines (LSmean ±SEM).

	Management routine			
	W1	W2	W3	P-value^a^
**Total number of sows**	14	13	16	
**All nursings**				
Nursing duration (s) (duration/7.5 h)	1338 ±220	1549 ±218	1329 ±218	n.s.
Nursing frequency (nursings/7.5 h)	8.5 ±0.8	8.0 ±0.8	7.8 ±0.8	n.s.
Duration per nursing (s)	121 ±47	207 ±43	223 ±49	n.s.
**Sow-terminated nursings**				
Nursing duration (s) (duration/7.5 h)	1102 ±147	1055 ±147	1059 ±144	n.s.
Nursing frequency of sow terminated nursings (nursing/7.5 h)	7.5 ±1.0	6.8 ±1.0	7.1 ±1.0	n.s.
Duration per nursing of sow terminated nursings (s)	130 ±14	146 ±14	144 ±14	n.s.
**Piglet-terminated nursings**				
Nursing duration (s) (duration/7.5 h)	542 ±247	802 ±241	568 ±254	n.s.
Nursing frequency of piglet terminated nursings (nursings/7.5 h)	1.7 ±0.4	1.8 ±0.4	1.6 ±0.4	n.s.
Duration per nursing of piglet terminated nursings (s)	392 ±68	482 ±64	400 ±68	n.s.

^a^ n.s. not significant

**Fig 1:**
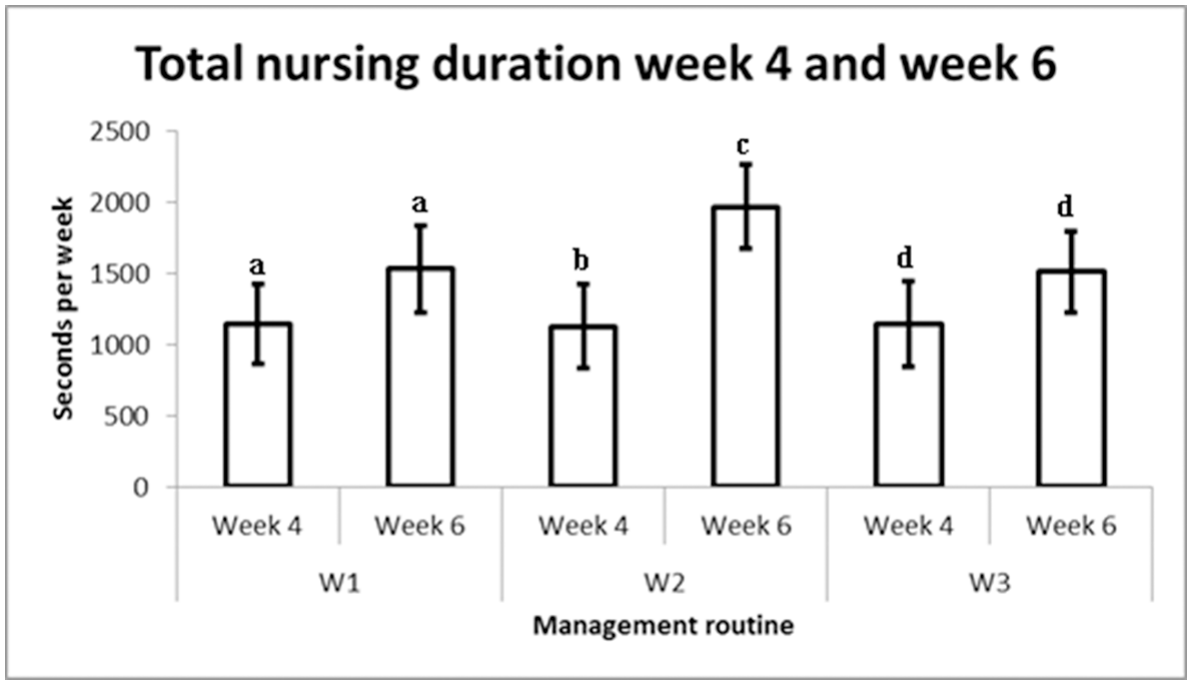
Total nursing duration (LSmean ±SEM) in week 4 and week 6 for the three management routines. Different letters indicate within management routine differences (*P*<0.05).

There were no significant differences between the two weeks of recording (weeks 4 and 6 of lactation) with respect to total nursing duration per day, nursing frequency per day, or duration per nursing event. However there was a pair-wise difference within management routine W2, where total nursing duration was longer in week 6 than week 4 (*P*<0.05) (Fig 1) and sow-terminated nursings were also longer in week 6 (1548 s/7.5 h ± 206) than week 4 (564 s/7.5 h ± 206) (*P*<0.001). The frequency of sow terminated nursings was higher in week 6 than week 4 for management routine W2 (8.5/7.5 h ± 1.3 vs. 5.0/7.5 h ± 1.3) (*P*<0.05). Duration per nursing for sow-terminated nursings was longer in week 6 than week 4 for management routine W2 (180s/7.5 h ± 24 vs. 122s/7.5 h ± 24) (*P*<0.001).

### Piglet performance

There were no differences between management routines for mean piglet weight at weaning, within-litter weight variation at weaning, or mean piglet weight gain during the nursing period (Table 3). There were also no differences in total litter weight at weaning or total litter weight gain from birth until weaning when comparing the three management routines (Table 3). There were no differences between the reference group at weaning and management routines at the corresponding time (week 5 post-farrowing) for mean piglet weight, within-litter weight variation, and total litter weight.

**Table 3:** Piglet performance parameters except piglet mortality for the three management routines (LSmean ±SEM).

	Management routine			
	W1	W2	W3	P-value^a^
Total number of litters	14	13	16	
Piglet weight at weaning (kg/piglet)	14.1±0.4	13.7±0.4	14.3±0.4	n.s.
Within-litter weight variation at weaning (SD, kg)	2.3±0.2	2.6±0.2	2.3±0.2	n.s.
Piglet weight gain (farrowing-weaning) (kg/piglet)	12.7±0.4	12.2±0.4	12.8±0.4	n.s.
Litter weight at weaning (kg/litter)	133.8±8.3	133.6±8.8	130.9±8.3	n.s.
Litter weight gain(kg/litter)	114.4±8.3	114.2±8.8	111.5±8.3	n.s.

^a^ n.s. not significant

**Fig 2:**
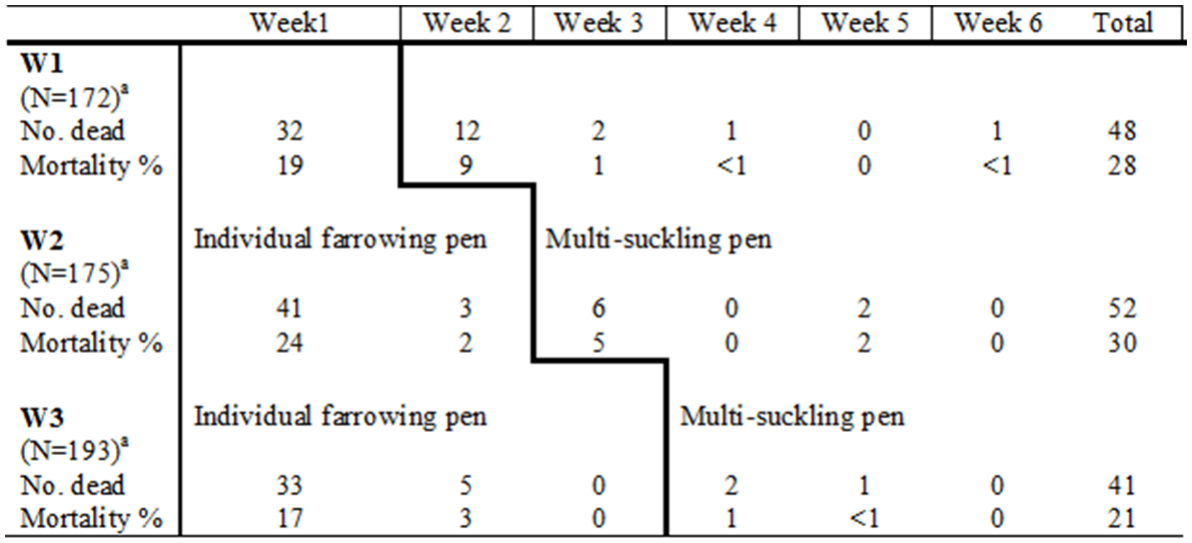
Piglet mortality per week from farrowing until weaning (week 6) for the three management routines. **Descriptive statistics.** ^a^ Piglets born alive within each management routine.

There was no difference in piglet mortality when the sow and piglets were housed in the farrowing pen (Fig 2). The mortality in the multi-suckling pen was higher for W1 than W2 (*P*<0.05) and W3 (*P*<0.05) (Table 4). There was no difference in piglet mortality between the reference group at weaning and management routines at the corresponding time (week 5 post-farrowing).

**Table 4:** Piglet mortality (%) (LSmean ±SEM) on sow level for the three management routines.

	Management routine			
	W1	W2	W3	P-value
Total number of sows	14	13	16	
Total mortality (%)	27.1±3.7	24.1±4.1	19.8±3.6	n.s
In the individual farrowing pen (%)	17.3±3.5	19.8±3.9	18.1±3.4	n.s
In the multi-suckling pen (%)	12.0±2.0^a^	5.8±2.2^b^	1.7±1.9^b^	0.05

^a, b^ different superscripts within rows indicate a significant difference

### Post-mortem findings in piglets from the multi-suckling pen

Three of the 16 piglets that died in multi-suckling pen in W1 were euthanized due to trauma or general weakness. Ten of the 16 piglets were considered by the barn staff to have died due to crushing. Seven of those 10 were considered thin on examination and four had no stomach contents. Three piglets were missing at examination.

Three of the eight piglets that died in the multi-suckling pen in W2 were euthanized due to general weakness. Three of the remaining five piglets were considered by the staff to have died due to crushing. None of the five piglets was considered thin and all had stomach contents.

Two of the three piglets that died in the multi-suckling pen in W3 were euthanized due to suspected infection (exudative epidermitis). One of these euthanized piglets was considered thin and did not have any stomach contents.

### Correlations between nursing-suckling interaction and piglet performance

Only biologically interesting and statistical significant correlations related to the aim of this study are presented below.

Overall, litter size at the time of group housing was negatively correlated to total mortality (r = -0.82: *P*<0.0001) and positively correlated to total litter weight at weaning (r = 0.73: *P*<0.0001). Overall, mortality in the multi-suckling pen regardless of management routine was positively correlated to total mortality (r = 0.51: *P*<0.001).

Duration per nursing event for sow-terminated nursings on both recording days within week 6 was positively correlated with total litter weight at weaning for W1 (r = 0.94: *P*<0.001; r = 0.91: *P*<0.05), but not for W2 and W3. Duration per nursing for sow-terminated nursings for both recording days within week 6 was positively correlated with litter size at weaning for W1 (r = 0.90: *P*<0.05; r = 0.96: *P*<0.05), but not for W2 and W3.

Nursing frequency of piglet-terminated nursings was positively correlated to within-litter weight variation at weaning for one of the recording days within week 6 for W2 (r = 0.99: *P*<0.05) and W3 (r = 0.96: *P*<0.05). Duration per nursing event for sow-terminated nursing was negatively correlated to within-litter weight variation at weaning for the first recording day within week 6 for W2 (r = -0.99: *P*<0.05).

The correlation between mortality in the multi-suckling pen and total mortality was positive for W1 (r = 0.61: *P*<0.05) and W2 (r = 0.62: *P*<0.05), but not for W3.

## Discussion

This study investigated whether nursing-suckling interaction and piglet performance differed between three management routines whereby lactating sows with piglets were group housed at one week (W1), two weeks (W2), or three weeks (W3) post-farrowing. Piglet performance was also compared with that in a reference group of individually loose-housed sows with piglets.

The majority of the piglets that died did so during the first week post-farrowing, which is in agreement with previous studies on piglet mortality in individual farrowing pens [8]. Total mortality in the multi-suckling pen in the present study was comparable to multi-suckling pen mortality in a previous study (0–12.1%) where piglets were weaned between five and six weeks of age [18]. For management routine W2 and W3 the mortality in the multi-suckling pen was significantly lower than W1. However, the mortality in the multi-suckling pen when group housing commenced at three weeks post-farrowing was not associated with total mortality, in contrast to when group housing commenced at one or two weeks post-farrowing. This is probably because three-week-old piglets are more robust than younger piglets at the start of group housing and therefore have a better chance of surviving in the multi-suckling pen. In addition, the number of piglets that died the last three weeks of group housing differed by only one piglet between management routines, which demonstrates low piglet mortality from three weeks post-farrowing (Fig 2). These results suggest that group housing of piglets at three weeks post-farrowing is more favorable, although the overall mortality did not differ significantly between management routines.

In management routine W2, total nursing duration was longer in week 6 than week 4, which might be explained by longer duration per nursing event for sow-terminated nursings and a higher frequency of sow-terminated nursings in week 6. Longer duration late in lactation was the opposite of the expected trend, as previous studies have shown decreased or unchanged nursing duration as the lactation period progresses [5, 19]. The higher frequency of sow-terminated nursings during week 6 than week 4 in W2 is in agreement with previous studies [4, 5, 19]. The results indicate that even though the sows in the present study did not have any additional space compared with ordinary organic production, e.g., outdoor access, the sows still carried out a continuous weaning process by terminating more nursings in late lactation.

Mean piglet weight in the litter at weaning did not differ significantly between management routines. The environmental change to which piglets are subjected when they are moved from the individual farrowing pen to the multi-suckling pen has previously been reported to negatively affect piglet growth, and thus piglet weaning weight [21–23]. It can be speculated that the positive correlation in week 6 within W1 between duration per nursing event for sow-terminated nursings and total litter weight at weaning/litter size at weaning indicates that growth and survival of W1 piglets in late lactation were more dependent on the sow’s willingness to nurse than solid feed consumption. In contrast, the piglets within W2 and W3 were older and had a more mature digestive tract to consume solid feed at the start of the group housing period, and were thus perhaps not as dependent on milk consumption for growth and survival in late lactation.

Except for piglet mortality, piglet performance parameters did not differ between management routines, or between each management routine and the reference group. It was not possible to investigate the nursing-suckling interaction during the time in the individual farrowing pens in this study, and therefore a comparison between the housing systems could not be made, which would have been preferable [24]. However, the results obtained here are in agreement with previous findings of no difference in pre-weaning piglet performance between piglets housed in individual farrowing pens and multi-suckling pens [9].

In general, within-litter weight variation increases with increasing piglet age [4, 6, 25]. The within-litter weight variation is perhaps more noticeable in organic piglet than in conventional piglet production simply because of the prolonged lactation period. Moreover, factors such as the sibling competition at nursing [26], partially covered teats due deep straw bedding [27], and decreased space at the udder with increasing piglet age could limit piglets’ teat access and therefore probably affect the within-litter weight variation at weaning.

The positive correlation between within-litter weight variation and the frequency of piglet- terminated nursings in W2 and W3 could perhaps be a result of variation between piglets in performing udder stimulation at nursing. A previous study has shown that the udder stimulation performed by the piglet post milk let-down increases milk production in that specific udder compartment [25], and that the will to massage differs between piglets [28]. Therefore when piglets terminate a high number of nursings the difference in the will to massage and its effect on milk production contributes to within-litter weight variation.

### Conclusions

There was no effect of management routine, i.e., group housing at one (W1), two (W2), or three weeks (W3) post-farrowing, on nursing-suckling interaction, piglet growth, and within-litter weight variation at weaning. However, group housing of sows and piglets three weeks post-farrowing was associated with lower piglet mortality in the multi-suckling pen than group housing at one week post-farrowing. Therefore group housing of sows and piglets at three weeks post farrowing appears most favorable as regards piglet survival.
